# Junctional tachycardia due to multisystem inflammatory syndrome in children with SARS-CoV-2 infection in a 12-year-old female

**DOI:** 10.1017/S1047951120005016

**Published:** 2021-01-07

**Authors:** Jaikaran Man Singh, Rachel L. Palting, Andras Bratincsak

**Affiliations:** 1 Department of Pediatrics, John A. Burns School of Medicine, University of Hawai‘i, Honolulu, Hawai‘i; 2 Hawai‘i Pacific Health Medical Group, Hawai‘i Pacific Health, Honolulu, Hawaii

**Keywords:** MIS-C, intravenous immunoglobulin, junctional tachycardia

## Abstract

A 12-year-old girl presented with fever and signs of systemic inflammation, and was found to have junctional tachycardia. She was subsequently diagnosed with Multisystem Inflammatory Syndrome in Children and treated with intravenous immunoglobulin and steroids, which led to resolution of the arrhythmia.

Multisystem Inflammatory Syndrome in Children can present with numerous cardiac manifestations including several types of arrhythmias and atrioventricular block, but junctional tachycardia has not been described until now.

## Case

A 12-year-old female, with no known medical condition, presented with a 4-day history of fever, myalgia, abdominal pain, vomiting, non-bloody diarrhoea, bilateral eye redness, and 1-day history of a diffuse erythematous rash. The patient had no known COVID-19 exposures and denied interacting with anyone else outside her household.

Upon presentation to the emergency department, the patient’s vital signs were notable for fever of 103.2 F, heart rate of 132 bpm, and lowest BP of 84/59 mmHg. On examination, she was found to have a conjunctival injection with limbic sparing as well as a non-blanching, erythematous confluent rash on her back, arms, and abdomen. Cardiac examination was significant for tachycardia, but no murmur, no gallop, or additional sounds were heard. Laboratory results were significant for lymphopenia and thrombocytopenia, as well as elevated procalcitonin (18.47 ng/ml), C-reactive protein (151.8 mg/L), and ferritin (391 ng/ml). She had mild hypokalaemia (3.1 mmol/L), but her electrolytes were otherwise normal. Chest X-ray noted a left perihilar infiltrate. Nasopharyngeal SARS-CoV-2 polymerase chain reaction was negative and Immunoglobulin G serum level was obtained. She was admitted to the Pediatric Intensive Care Unit with concerns of septic shock or toxic shock syndrome, and started on ceftriaxone and vancomycin.

**Figure 1.**
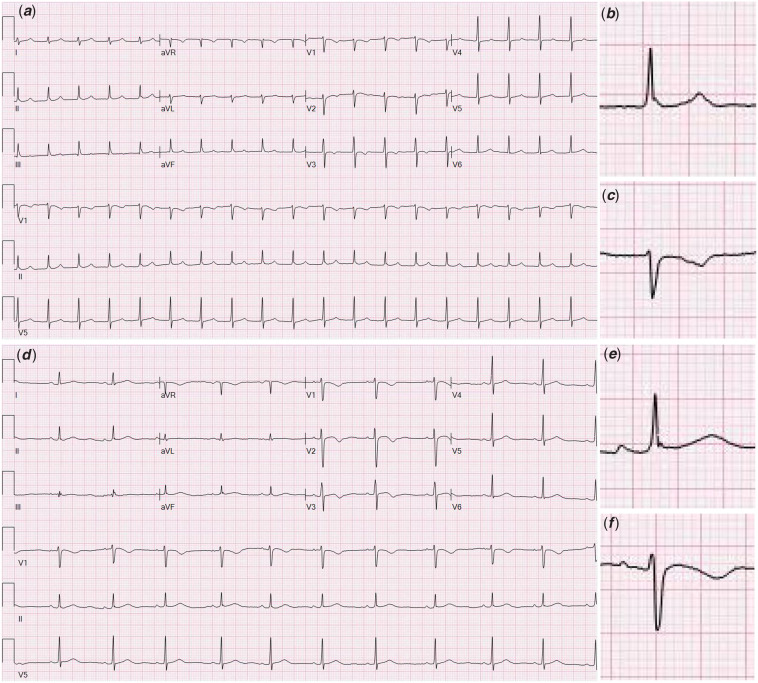
Electrocardiograms showing junctional tachycardia (a) with retrograde P waves visible in inserts of lead II (b) and V1 (c), and normal sinus rhythm (d) with normal P waves preceding the QRS visible in inserts of lead II (e) and V1 (f).

Soon after admission, she started complaining of chest pain, which prompted further cardiac workup and increased concern for MIS-C. Electrocardiogram and telemetry were significant for junctional tachycardia (Fig 1) with a maximum heart rate of 132 bpm. Troponin T was elevated at 49 ng/L and pro-B-type natriuretic peptide was 4544 pg/ml. A repeat potassium level improved to 3.7 mmol/L without intervention. Initial echocardiogram demonstrated significantly decreased left ventricular ejection fraction of 42–50%. However, her blood pressures remained stable and she did not require any inotrope support. Based on elevated cardiac enzymes and decreased left ventricular function in association with clinical signs, the patient was diagnosed with MIS-C, and she was started on intravenous immunoglobulin 2 g/kg and solumedrol 2 mg/kg. SARS-CoV-2 immunoglobulin G test collected on hospital day 1 was elevated, confirming the diagnosis of MIS-C.

Over the subsequent 12 hours, her junctional tachycardia resolved with the return of normal sinus rhythm. Electrocardiogram was repeated after completion of the first intravenous immunoglobulin infusion and showed normal sinus rhythm. Repeat echocardiogram showed improvement of left ventricular ejection fraction, but with mild diffuse dilation of the right coronary artery and left anterior descending artery (z scores of 2.52 and 2.63, respectively). She was started on aspirin 81 mg daily due to coronary artery dilation. She clinically improved over the course of her hospitalisation, and was discharged home after 5 days on steroid taper and aspirin. Electrocardiogram on the day of discharge showed normal sinus rhythm and echocardiogram showed normal left ventricular function (left ventricular ejection fraction 60%) and complete resolution of coronary artery dilation. Troponin T on the day of discharge was 52 ng/L.

The final diagnosis was MIS-C associated with SARS-Cov-2 infection and cardiac involvement with left ventricular dysfunction due to autoimmune myocarditis, coronary artery dilation, and junctional tachycardia.

On follow-up in our cardiology clinic 1 week after discharge, she reported no recurrence of symptoms and continued to have a completely normal echocardiogram and electrocardiogram.

## Discussion

Multisystem Inflammatory Syndrome in Children is a complication of SARS-CoV-2 first described in the United Kingdom in April, 2020.^1^ Whereas, acute COVID-19 presents within 2–3 days of infection and is likely due to direct viral injury, MIS-C usually presents 2–4 weeks after the initial infection and is due to an abnormal immune response.^2^ It presents as a hyperinflammatory syndrome involving multiple organs of the body. The heart is commonly involved with some degree of ventricular dysfunction being seen in about 50% of patients, and coronary artery dilation is seen in about 25% of patients.^3^ In a review of cardiac manifestations of MIS-C.^3^ numerous arrhythmias were documented including atrial fibrillation, premature ventricular contractions, and QT prolongation. There were also case reports of various degrees of atrioventricular block and junctional escape rhythm.^4,5^ however, there have been no documented cases of junctional tachycardia. The arrhythmias described in acute COVID-19 infection in adults are somewhat different, and may include atrial fibrillation, supraventricular tachycardia, and ventricular tachycardia besides first-degree AV block.^6^ Notably, arrhythmias in adults are usually diagnosed during the acute infection, however, arrhythmias in children are associated with MIS-C, occurring 2–4 weeks after the initial infection.

In this patient, the initial electrocardiogram showed junctional tachycardia with the absence of consistent P waves preceding the QRS complexes (Fig 1). She had no prior history of arrhythmia or cardiac symptoms and none of her concurrent medications have any known association with junctional tachycardia. Her arrhythmia resolved within 12 hours after the initiation of recommended treatment for MIS-C: intravenous immunoglobulin and steroids.^7^ Given the rapid resolution of her arrhythmia with the anti-inflammatory medications, the junctional tachycardia was due to carditis induced by MIS-C. The mechanism of junctional tachycardia in MIS-C is likely secondary to inflammation of the atrioventricular node, similar to the inflammation and dysfunction of the conduction system seen in myocarditis, acute rheumatic fever, and lyme carditis.^8^

Based on our experience, junctional tachycardia should be included amongst the various arrhythmias and cardiac manifestations of MIS-C. There is no antiarrhythmic medication required beyond the usual anti-inflammatory treatment recommended for MIS-C.

## Conclusion

Multisystem Inflammatory Syndrome in Children may present with junctional tachycardia, and can successfully be treated with intravenous immunoglobulin and steroids.
